# Factors Influencing Health Equity of Influenza Vaccination in Pediatric Patients

**DOI:** 10.1097/pq9.0000000000000543

**Published:** 2022-03-30

**Authors:** Lane F. Donnelly, Matthew Wood, Jean Chantra, Ling Loh, Brendan Burkart, Grace M. Lee

**Affiliations:** From the 1 Center for Pediatric and Maternal Value, Lucile Packard Children’s Hospital – Stanford, Stanford Children’s Health, Palo Alto, Calif.; 2 Department of Pediatrics, School of Medicine, Stanford University, Palo Alto, Calif.; 3 Department of Radiology, School of Medicine, Stanford University, Palo Alto, Calif.

## Abstract

Social factors can be a determinate for multiple health outcomes. We evaluated the association of numerous social factors on rates of influenza nonvaccination in a large pediatric primary and subspecialty care system.

**Methods:**

During the 2019–2020 influenza vaccination season, we calculated the nonvaccination rate for a pediatric healthcare system with both subspecialty and primary care practices. We compared influenza vaccination rates for factors that might affect health equity (patient gender, language preference, health insurance payer category, race and ethnicity, and estimated median household income based on zip code analysis) by creating simultaneous 95% confidence intervals using the Wilson method with continuity correction and a Bonferroni adjustment for the number of categories compared.

**Results:**

The overall influenza nonvaccination rate was 58.0% (59,375 not vaccinated of 102,377). Statistically significant differences in nonvaccination rate were present for the following health equity indicators: Spanish (75.6%) and Chinese Dialects (78.0%) > English (55.9%) speaking; Hispanic (70.1%) > many other race and ethnicities; Asian (51%) < many other race and ethnicities; Commercial (53.5%) < Public (71.2%) or Self (81.4%) pay; and lower (67.6%–79.1%) > higher median household income (52.9%–56.4%).

**Conclusions:**

Non-English language preference, Hispanic ethnicity, public insurance, and lower median household income are associated with a decreased likelihood of influenza vaccination. We are using these data to inform our key drivers to improve influenza vaccination in our system.

## INTRODUCTION

Influenza can be associated with significant illness, hospitalization, and death in children.^1–3^ Children can also serve as vectors in spreading disease to at-risk adults.^4^ Vaccination of children is an effective way to prevent illness and the spread of influenza. Yearly influenza vaccination is recommended for all children aged greater than 6 months, by the US Advisory Committee on Immunization Practices and the American Academy of Pediatrics.^5,6^ Despite these recommendations, national influenza vaccination rates for children remain below targets.^1,2,7,8^

A growing literature shows that several social factors can predict health outcomes. Higher infant mortality,^9^ worse outcomes after pediatric surgery,^10^ and longer wait times for kidney transplantation^11^ have been observed in African American and Hispanic children compared with White children. Previous survey-based studies show variable disparities in influenza vaccination rates for Hispanic and African American children.^1,12–14^ Studies also show that children in rural areas are less likely to receive influenza vaccination than those in urban areas.^15^ Less has been published about the influence of other social factors on influenza vaccination in children. In this health equity initiative, we evaluated the association of multiple factors that might impact influenza vaccination rates in a pediatric healthcare system that delivers primary and subspecialty care to our pediatric populations. We performed a causal analysis (fishbone diagram) for factors contributing to potential health inequities in influenza vaccination rates. We used the inequity data and causal analysis to create a key driver diagram for improvement. By understanding quality and safety outcomes through the lens of health equity, we identify interventions to overcome barriers to vaccination and close the quality gap.

## MATERIALS AND METHODS

Our healthcare system monitors data on key performance indicators as part of our institutional oversight and continuous improvement of quality, safety, and service. We have now begun evaluating the differential performance of each of these key performance indicators by factors influencing health equity. Influenza vaccination rate is one of the key performance indicators tracked. Following the guidelines created by our institutional improvement center and our institutional review board, this project met criteria as quality activity, was not considered human subjects research, and was exempt from review by our institutional review board.

The influenza vaccination season was evaluated from September 16, 2019 through April 30, 2020. Our pediatric health system consists of a quaternary children’s hospital, multiple pediatric subspecialty clinics at various locations, and a pediatric primary care network. For this study, we evaluated the influenza nonvaccination rate. This was chosen in contrast to the vaccination rate, as the quality focuses on minimizing the number of children not vaccinated. The nonvaccination rate was calculated by dividing the number of patients not vaccinated for influenza by the total unique eligible patients. Eligible patients included all patients with an outpatient clinic visit (or multiple) during the influenza vaccination season who were aged at least 6 months and less than 22 years old at the time of their visits. This included both in-person and virtual video visits. Our pediatric health care system does not have an emergency department (it is run by our adult health care system academic partner); so emergency visits were not included in the denominator. Our pediatric health care system does not have free-standing pediatric urgent care centers. However, our primary care providers have off-hour urgent walk-in clinics. Such visits were included in the denominator. Our health system offers influenza vaccination at multiple sites and in-person influenza vaccination clinics. We considered a patient vaccinated if they received the vaccination from our healthcare system or elsewhere. Vaccination external to our system was documented through a combination of asking the family and documenting in our electronic health record and checking the California Immunization Registry [cairweb.org]. Patients who were offered the vaccination but declined were considered not vaccinated.

We evaluated differences in the influenza nonvaccination rate based on the following factors: patient gender, language preference, health insurance payer category, race, and ethnicity, and estimated median household income based on zip code analysis. We collected all data from our electronic health records. Such data are recorded into our electronic health record at registration, and all historic entries were evaluated for each factor. If the same value was consistently documented through time (same response at each encounter), it was assigned that value for that particular demographic factor. If the value was inconsistently documented over time (different responses at different encounters), it was categorized as “other” for that specific demographic factor.

Patient gender was masculine, feminine, or other/unknown. Race and ethnicity were evaluated by combining the race field, ethnicity field, and a multiracial flag in our electronic health system. If the flag for multiracial was selected, the patient was considered multiracial. Otherwise, if the patient selected Hispanic as their ethnicity, they were categorized as Hispanic. If the patient was not multiracial or Hispanic, they were classified as the race they selected. Options for race included White, Asian, Black or African American, Native Hawaiian or other Pacific Islander, or Other. If they declined to answer the question, they were categorized as declined. If there were no data in the ethnicity and race fields, the patient was classified as unknown.

For language preference, patients were categorized as speaking English, Spanish, Chinese (we combined various dialects such as Mandarin, Cantonese, and others). At registration, the patient and family are asked their language of preference, and that information is recorded into the electronic health record.

For health insurance payer type, patients were categorized as commercial insurance, public insurance (Medicaid), or self-pay, using a methodology similar to that utilized by US News & World Report Ranking of Children’s Hospitals methodology.^16^ The percentages for various payers were calculated by evaluating the number of patients with that type of insurance divided by the total number of patients. This is opposed to calculating the total charges per insurance type, as is done when creating the payer mix often displayed in finance evaluations. If the primary payer was ever commercial insurance during any visit, the patient was categorized as commercial. Otherwise, if the primary payer was public insurance during all visits, the patient was categorized as public. If the patient was self-pay during every visit, the patient was considered self-pay.

For each of our unique patients, we determined the zip code for their home residence. The zip code was unavailable in 0.01% of all patients, and 21% had multiple zip codes over time; so their median household income was not determined. For patients originating in California, which was 78% of the patients seen in the study, we categorized each patient with the median household income for their residential zip code. The median household income was based on the 2019 American Community Survey.^17^ Median household income was categorized as $0–50,000; $50,000–100,000; $100,000–150,000; $150,000–200,000; or $200,000 and above.

To compare influenza vaccination rates by gender, race/ethnicity, language preference, insurance status, and estimated median household income, simultaneous 95% confidence intervals were calculated using the Wilson method with continuity correction and a Bonferroni adjustment for the number of categories compared. Statistical significance was defined as a *P*-value < 0.05. The evaluation was performed using R.^18^

**Fig. 1.**
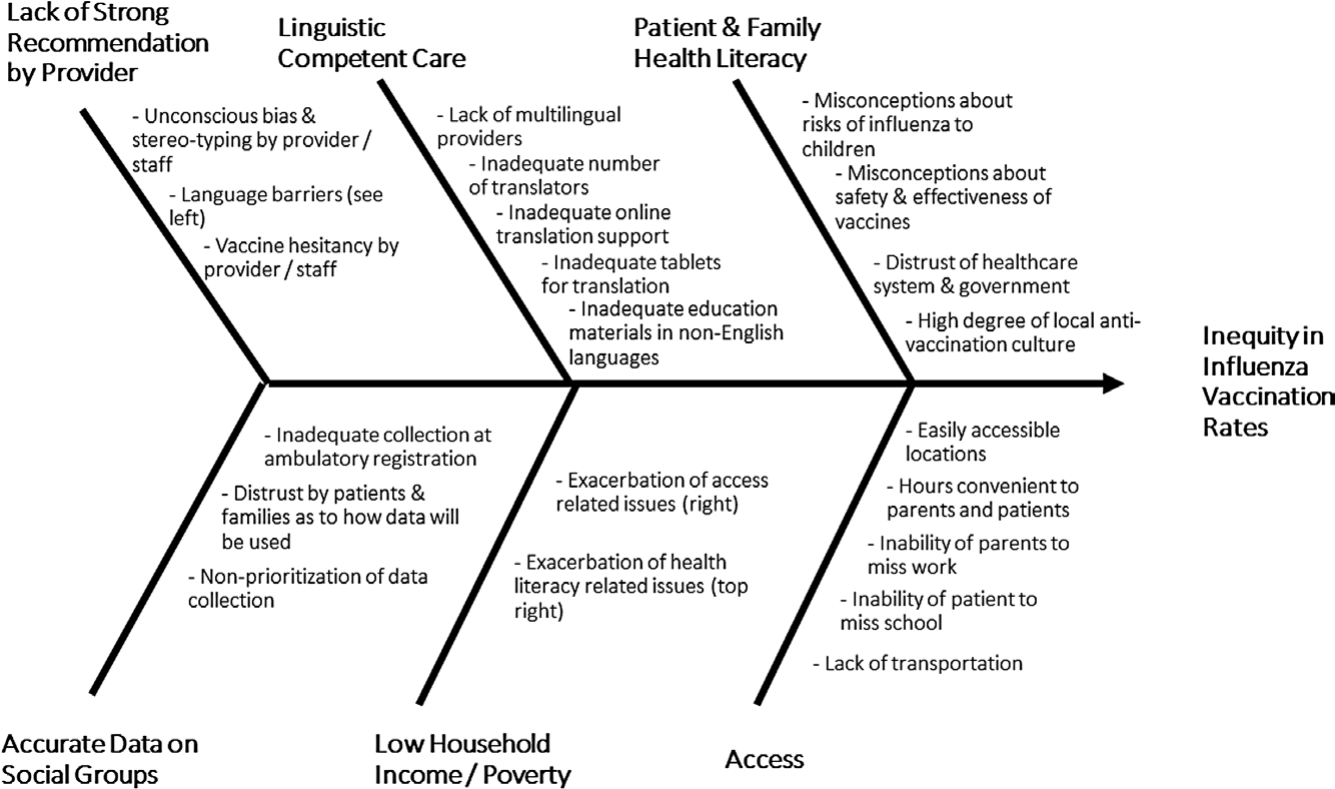
Causal analysis (fishbone diagram) showing factors potentially contributing to inequity of pediatric influenza vaccination rates.

We performed a causal analysis to look for factors in our system potentially contributing to any identified disparities in influenza vaccination rates identified in the analysis described above. This information was displayed in a fishbone diagram (Fig. 1).

**Fig. 2.**
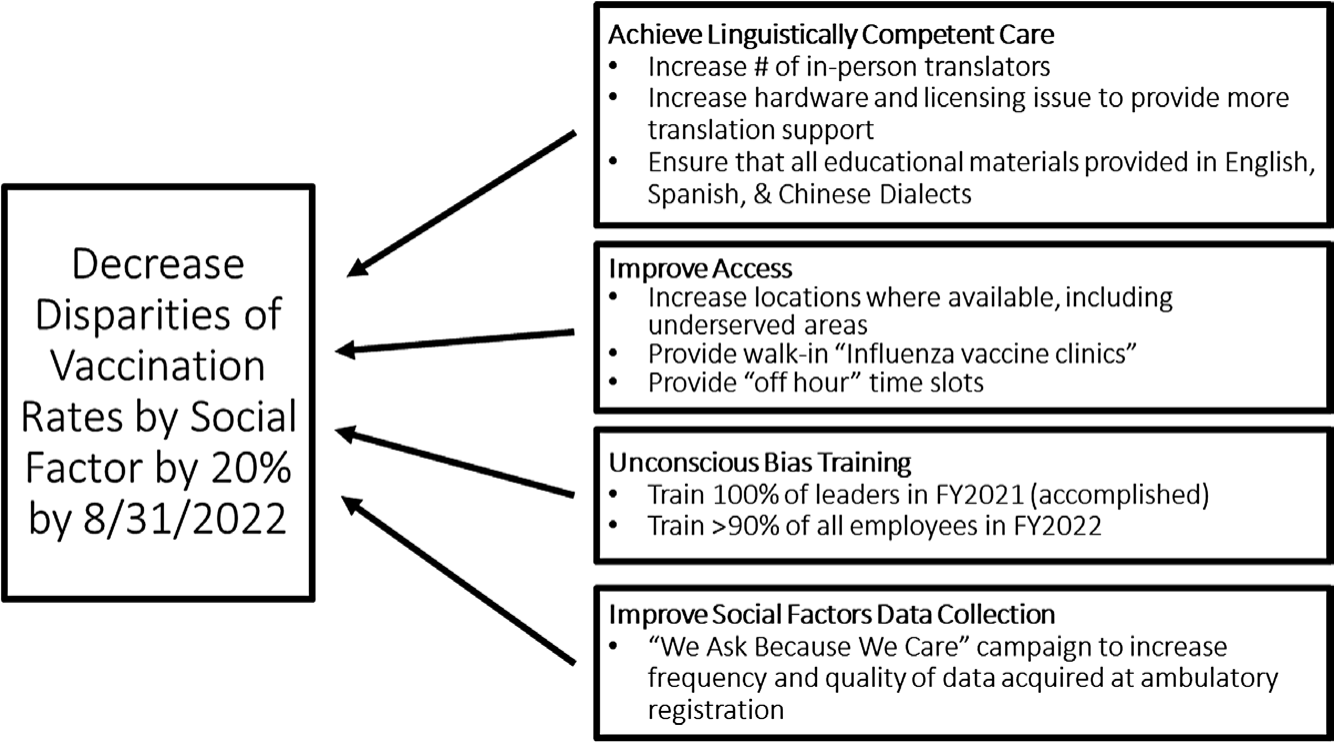
Key drivers to decrease disparities in vaccination rates by social factors.

The data concerning discrepancies in influenza vaccination and the results of the causal analysis were used to create a key driver diagram (Fig. 2) outlining the plan to reduce inequities in the influenza vaccination rate for the upcoming influenza vaccination rate season (Fall 2021–Spring 2022).

## RESULTS

During the 2019–2020 influenza vaccination period, there were encounters with 102,377 unique pediatric patients. Of those, 59,375 were not vaccinated. This results in an overall novaccination rate of 58.0%. This compares to a nonvaccination rate of 63% in 2018–2019.

Table 1 shows the demographics for patients seen during the study period. Age ranged through the spectrum of pediatric patients with a mean age of 9.23 years and a median of 9.18 years. Importantly, in 26% of patients, Race & Ethnicity were unknown. Less than 0.01% of patients (10 patients) were unknown gender or documented as other than masculine or feminine.

**Table 1.** Demographics by Health Equity Indicator for Pediatric Patients Seen during the 2019–2020 Influenza Vaccination Season

Parameter	No. Patients in Category	% of Patients in that Parameter
Total	102,377	100.0%
Gender		
Feminine	49,464	48.3%
Masculine	52,903	51.7%
Language Preference		
English	90,491	88.4%
Spanish	9367	9.1%
Chinese dialects	1036	1.0%
Other	1483	1.4%
Payer		
Commercial	76,417	74.6%
Public	25,750	25.2%
Self-Pay	210	0.2%
Race & ethnicity		
Unknown	26,669	26.0%
White	24,125	23.6%
Hispanic	17,868	17.5%
Asian	14,389	14.1%
Declines to State	7679	7.5%
Other	5877	5.7%
Multiracial	3321	3.2%
Black or African American	1888	1.8%
Native Hawaiian or Other Pacific Islander	460	0.4%
American Indian or Alaska Native	101	0.1%
California Median Household Income by Zip Code		
$0–50,000	1046	1.0%
$50,000–100,000	25,163	24.6%
$100,000–150,000	31,213	30.5%
$150,000–200,000	18,757	18.3%
$200,000 and above	3760	3.7%

Table 2 shows the number of patients not vaccinated and nonvaccination rates for each equity factor. Based on the simultaneous 95% confidence intervals, the following differences are statistically significant within each health equity factor. The nonvaccination rate for English-speaking patients (55.9%) was significantly less than for Chinese Dialect (78.0%) and Spanish-speaking patients (75.6%). The nonvaccination rate for Hispanic patients (70.1%) was significantly greater than for the following subcategories: Asian (50.5%), Native Hawaiian or Other Pacific Islander (56.3%), White (58.2%), Black or African American (61.4%), and Multiracial (55.6%). The nonvaccination rate for Asian patients (50.5%) was significantly lower than for White (58.2%), Black or African American (61.4%), Hispanic (70.1%), and Multiracial (55.6%). The nonvaccination rate for commercial payer (53.5%) was significantly lower than for public payer (71.2%) or self-pay (81.4%). The nonvaccination rate for public (71.2%) was significantly lower than for self-pay (81.4%). For median household income by zip code, the three highest income categories ($100,000–150,000, $150,000–200,000; or $200,000 and above) had nonvaccination rates (53.6%–56.4%) that were significantly lower than the lowest two income categories ($0–50,000; $50,000–100,000) (67.6%–79.1%).

**Table 2.** Influenza Nonvaccination Rates Broken Down by Categories in Various Health Equity Indicators

Parameter	No. Patients Not Vaccinated	Influenza Nonvaccination Rate	95% Confidence Interval
Gender			
Masculine	30,817	58.3%	(57.8%, 58.7%)
Feminine	28,555	57.7%	(57.2%, 58.2%)
Language			
Chinese dialect	808	78.0%	(74.6%, 81.1%)*
Spanish	7082	75.6%	(74.5%, 76.7%)*
Other	923	62.2%	(59.0%, 65.4%)
English	50,562	55.9%	(55.5%, 56.3%)*
Payor			
Self-pay	171	81.4%	(73.9%, 87.2%)*
Public	18,327	71.2%	(70.5%, 71.8%)*
Commercial	40,877	53.5%	(53.1%, 53.9%)*
Race & ethnicity			
Hispanic	12,527	70.1%	(69.1%, 71.1%)*
American Indian or Alaska Native	65	64.4%	(49.9%, 76.7%)
Black or African American	1160	61.4%	(58.2%, 64.6%)
White	14,047	58.2%	(57.3%, 59.1%)
Native Hawaiian or Other Pacific Islander	259	56.3%	(49.7%, 62.7%)
Unknown	14,846	55.7%	(54.8%, 56.5%)
Multiracial	1846	55.6%	(53.1%, 58.0%)
Declines to State	4221	55.0%	(53.4%, 56.6%)
Other	3139	53.4%	(51.6%, 55.2%)
Asian	7265	50.5%	(49.3%, 51.7%)*
Income			
$0–$50,000	827	79.1%	(75.6%, 82.2%)*
$50,000–$100,000	17,006	67.6%	(66.8%, 68.3%)*
$100,000–$150,000	17,603	56.4%	(55.7%, 57.1%)*
$150,000–$200,000	9921	52.9%	(51.9%, 53.8%)*
$200,000 and above	2017	53.6%	(51.5%, 55.7%)*

*Denotes statistically significant difference.

Table 3 shows the nonvaccination rate by age. Nonvaccination rates ranged from 42% in the youngest patients to 75% in the oldest patients.

**Table 3.** Nonvaccination Rate by Patient Age for Pediatric Patients Seen During the 2019–2020 Influenza Vaccination Season

Age at First Visit	Total Count	NOT Vaccinated	% NOT Vaccinated
6 m < X < 1	5965	2488	42%
1	6431	2897	45%
2	5869	2789	48%
3	5460	2811	51%
4	5361	2844	53%
5	5564	3105	56%
6	5105	2904	57%
7	5161	2938	57%
8	5155	2962	57%
9	5072	2980	59%
10	5207	3082	59%
11	5406	3237	60%
12	5551	3449	62%
13	5500	3481	63%
14	5599	3734	67%
15	5610	3734	67%
16	5308	3555	67%
17	4758	3254	68%
18	2230	1589	71%
19	1070	799	75%
20	609	454	75%
21	386	289	75%

### Causal Analysis

Results of the causal analysis are displayed in the fishbone diagram represented in Figure 1. The causal analysis and actions outlined on the key driver diagram derive from the work of multiple groups and committees, including the Diversity, Equity, and Inclusion Committee, Influenza Vaccination Committee, Family-Centered Care, and Ambulatory Quality Assurance and Performance Improvement Committee. These committees consist of quality and safety support personal, physician and nurse leadership, front line workers, and family members. The analysis included a literature review described below.

Much is written about the contributing factors at play regarding vaccination rates of Hispanic and African American children. Patients with considerable trust in their healthcare provider have improved health outcomes in multiple scenarios.^19,20^ A primary predictor of influenza vaccination rate includes a strong recommendation by a health care provider.^2^ Unconscious bias or minority stereotyping by providers and staff during patient interactions could lead to differences in whether a strong recommendation is made or not. Unconscious bias in health care has been well-documented and associated with health disparities.^1,21^ Issues related to access may also play a factor. This may include convenient vaccination locations, nonavailability of appointment times that fit the parent’s schedule, missed parental work, missed school, lack of transportation, and failure to seek care. Acceptance can also be a factor. Health literacy of the patient and family, such as misconceptions about the lack of risk related to influenza or vaccine effectiveness and safety, may also play a factor.^1,2^ Studies have shown that African American and Hispanic women are less confident in vaccine safety and efficacy and less likely to perceive the risk of acquiring vaccine-preventable diseases,^19^ and this may factor into decisions concerning vaccinating their children. African American women have a greater distrust of the government’s motives and are less likely to trust the safety of maternal or childhood immunizations in general.^17,22,23^ Such beliefs may affect the influenza vaccination rates of these mother’s children. Local differences in any of the above factors could explain differences between local or regional data concerning influenza vaccination and the aggregate national data.

Our study showed no difference in vaccination rate by gender in children. Imburgia^2^ showed no difference in vaccination rate by patient gender. Studies show that feminine gender has divergent predictive associations depending on the health outcome studies. For example, feminine gender has increased mortality compared with male patients for pediatric surgical procedures but decreased morbidity.^10^

To our knowledge, language preference has not been well studied as a potential health equity factor regarding influenza vaccination. We found that non-English speakers were more likely to not be vaccinated than English-speaking patients. Given the previously mentioned importance of a strong provider recommendation for vaccination having a positive effect on vaccination rates,^2^ it is not surprising that language barriers could harm influenza vaccination rates. Having linguistically competent care^1^ with multi-lingual healthcare providers, materials in pertinent non-English languages, as well as adequate translation support could have a positive effect on vaccination rates. As the patient population we serve is significantly Hispanic and non-English speaking, many of our initiatives involve improving linguistically competent care.

Our finding that a significantly greater influenza nonvaccination rate for public insurance compared with commercial insurance is also different from previous survey-based studies of national data.^1,2,6^ For example, Imburgia^2^ showed no statistically significant difference in vaccination rates based on insurance coverage.

We are not aware of previous publications examining the association between median household income by zip code and influenza nonvaccination rates. In the United States, zip code is one of the more accurate proxies for poverty.^17^ Our study showed a statistically significant association between lower household income by zip code and nonvaccination rate. Access and acceptance factors discussed above may play a role in the relationship between median household income and nonvaccination rate.

### Key Drivers for Improvement

We used a combination of the data showing discrepancies in influenza vaccination and the results of the causal analysis to create a key driver diagram (Fig. 2) with an aim to reduce inequities in the influenza vaccination rate for the upcoming influenza vaccination rate season (Fall 2021–Spring 2022). The four key drivers selected were: achieve linguistically competent care, improve access, unconscious bias training, and improve social factors data collection. As our system sees a significant number of non-English speaking patients, with Spanish being the most common, focused efforts to achieve linguistically competent care are one of our primary focuses. Multiple initiatives to increase access through adding influenza vaccination clinics and expanded hours have been implemented. Our organization is also undergoing unconscious bias training. We also have a campaign called “We Ask Because We Care” to improve the frequency of social factor data collection at registration.

## DISCUSSION

Our organization has made it an institutional priority to look at differential performance on key performance indicators in quality, safety, and service by factors that could influence health equity. We are using existing electronic health record data, and regular interval reports in our system to inform key interventions for quality improvement that can address such disparities. This particular evaluation focuses on social factors that may influence influenza vaccination rates in children.

Our local data show more pronounced disparities than some previously published national data showing a decrease in disparities in vaccination rates over time.^14,24,25^ Since the establishment of the Vaccines for Children Program (VFC) in 1994, national trends show decreased disparity-related vaccination rates for diphtheria-tetanus-acellular pertussis, measles-mumps-rubella, and polio vaccinations based on race, ethnicity, and income groups for.^19,20^ Other studies show decreases in disparity-related influenza vaccination rates based on race and ethnicity in children over the 2010–2016 time frame.^14^ National data can mask the existence of more pronounced regional or local disparities.

Most previous studies examining influenza vaccination rate predictors have been based on survey information.^1,2,14^ Our study was different because it looked at the actual influenza vaccination rate data for a pediatric healthcare system. Concerning race and ethnicity, those previous survey-based studies have shown various associations between race and ethnicity and vaccination rates. Consistent with our study, some have shown higher nonvaccination rates for Hispanic children.^1,2^ Others have shown lower rates of nonvaccination for Hispanic children than for other races and ethnicities.^8,12,14^ Other studies have often shown higher nonvaccination rates for African American children.^2,8,14^ Similarly, as seen in our research, other studies have shown lower nonvaccination rates in Asians.^8^

This work has multiple limitations. First, as a single healthcare system study, our work may not easily extrapolate to other organizations or populations. Specifically, our serviced population is diverse with significant White, Hispanic, and Asian populations, but African American patients represent only 2%. With these small numbers, it was unlikely that statistically significant trends or conclusions can be derived from our data concerning nonvaccination rates in the African American population. A second limitation is for 26% of patients, no race and ethnicity data were identified during the encounter. Third, we do not have data on which races and ethnicities may or may not be over represented in the “Unknown” group. Fourth, this work evaluates the combination of pediatric primary care and subspecialty pediatric practices, combined, looking at our entire ambulatory system, but does not address potential differences between primary and subspecialty care. Fifth, our estimates of nonvaccination rates may be conservative because patients received vaccines elsewhere and that data were not fully captured. We do not believe this is a significant factor because of both questions about vaccination being mandatory fields in our electronic health record for ambulatory visits and having access to the California Immunization Registry. Also, our declination rates are likely higher than some other geographic areas, given local cultural attitudes toward vaccinations in general. Finally, as our evaluation was based on electronic health record data, the patient had to have a healthcare encounter during the influenza vaccination period to be included in the assessment. Therefore, patients in our system who were not seen during that time period are not included in the rate calculations and may have been missed. This is a disadvantage of the electronic health record approach compared with survey data.

Due to the observational nature of the data and other limitations detailed previously, we chose to limit our study to focus individually on each factor’s relation to nonvaccination rates. Multivariate analysis could explore the extent to which each factor is confounded with the others. Such an analysis could be accomplished via a future study across organizations that appropriately stratify the observations across each of the categorical variables.

## CONCLUSION

In conclusion, in our pediatric healthcare system, multiple health equity factors (including non-English language preference, Hispanic race/ethnicity, public insurance payer, and lower household income) are associated with decreased likelihood of influenza vaccination. Better understanding of these contributing social factors and actions to decrease their influence has been factored into our key drivers to improve pediatric influenza vaccination rates and decrease disparities between groups with certain social factors in our system.

## DISCLOSURE

The authors do not have any financial interest to declare in relation to the content of this article.
